# A Multi-Cohort Study of Polymorphisms in the GH/IGF Axis and Physical Capability: The HALCyon Programme

**DOI:** 10.1371/journal.pone.0029883

**Published:** 2012-01-10

**Authors:** Tamuno Alfred, Yoav Ben-Shlomo, Rachel Cooper, Rebecca Hardy, Cyrus Cooper, Ian J. Deary, Tom R. Gaunt, David Gunnell, Sarah E. Harris, Meena Kumari, Richard M. Martin, Avan Aihie Sayer, John M. Starr, Diana Kuh, Ian N. M. Day, the HALCyon study team

**Affiliations:** 1 School of Social and Community Medicine, University of Bristol, Bristol, United Kingdom; 2 MRC Unit for Lifelong Health and Ageing and Division of Population Health, University College London, London, United Kingdom; 3 MRC Lifecourse Epidemiology Unit, University of Southampton, Southampton, United Kingdom; 4 Institute of Musculoskeletal Sciences, University of Oxford, Oxford, United Kingdom; 5 Centre for Cognitive Ageing and Cognitive Epidemiology, University of Edinburgh, Edinburgh, United Kingdom; 6 Department of Psychology, University of Edinburgh, Edinburgh, United Kingdom; 7 MRC Centre for Causal Analyses in Translational Epidemiology, School of Social and Community Medicine, University of Bristol, Bristol, United Kingdom; 8 Medical Genetics Section, University of Edinburgh, Edinburgh, United Kingdom; 9 Department of Epidemiology and Public Health, University College London, London, United Kingdom; 10 Academic Geriatric Medicine, University of Southampton, Southampton General Hospital, Southampton, United Kingdom; 11 Geriatric Medicine Unit, University of Edinburgh, Royal Victoria Hospital, Edinburgh, United Kingdom; Memorial Sloan Kettering Cancer Center, United States of America

## Abstract

**Background:**

Low muscle mass and function have been associated with poorer indicators of physical capability in older people, which are in-turn associated with increased mortality rates. The growth hormone/insulin-like growth factor (GH/IGF) axis is involved in muscle function and genetic variants in genes in the axis may influence measures of physical capability.

**Methods:**

As part of the Healthy Ageing across the Life Course (HALCyon) programme, men and women from seven UK cohorts aged between 52 and 90 years old were genotyped for six polymorphisms: rs35767 (*IGF1*), rs7127900 (*IGF2*), rs2854744 (*IGFBP3*), rs2943641 (*IRS1*), rs2665802 (*GH1*) and the exon-3 deletion of *GHR*. The polymorphisms have previously been robustly associated with age-related traits or are potentially functional. Meta-analysis was used to pool within-study genotypic effects of the associations between the polymorphisms and four measures of physical capability: grip strength, timed walk or get up and go, chair rises and standing balance.

**Results:**

Few important associations were observed among the several tests. We found evidence that rs2665802 in *GH1* was associated with inability to balance for 5 s (pooled odds ratio per minor allele = 0.90, 95% CI: 0.82–0.98, p-value = 0.01, n = 10,748), after adjusting for age and sex. We found no evidence for other associations between the polymorphisms and physical capability traits.

**Conclusion:**

Our findings do not provide evidence for a substantial influence of these common polymorphisms in the GH/IGF axis on objectively measured physical capability levels in older adults.

## Introduction

Muscle mass declines with age [1], [2] and low muscle mass and function in older people have been associated with a range of adverse outcomes [3], including poorer mobility [4], increased disability [1], [2] and higher mortality rates [5]. Low muscle mass and area are also independently associated with lower levels of objective measures of physical capability, the capacity to undertake the physical tasks of daily living, including grip strength [6], ability to balance [7] and to complete five chair stands [7]. Therefore, understanding the contributors to the inter-individual variability of muscle properties is important and may be relevant to the prevention of the adverse consequences of lower levels of physical capability [8]–[10]. Variability attributed to the growth hormone/insulin-like growth factor (GH/IGF) axis may also have the potential to lead to the development of novel interventions [11].

The GH/IGF axis plays an important role in body growth and composition [12]. IGF-I is produced in the liver in response to GH, as well as in skeletal muscle, and plays an important role in muscle growth and function [11]–[15], promoting and differentiating muscle cells, together with IGF-II [13]. Of the six binding proteins, most of IGF-I is bound to IGFBP-3 [12], which regulates the bioactivity of IGF-I and is expressed in skeletal muscle [14], possibly having IGF-I independent effects. Insulin receptor substrate 1 (IRS-1) interacts with the IGF receptor [14] and is involved in regulating body size [16]. The GH receptor is expressed throughout the body [12] and knockout of the *GHR* gene in mice leads to a reduction in body and muscle mass [17]. In addition to muscle function, the GH/IGF axis is also believed to play a role in some cancers [16], [18], longevity and ageing [19]–[21]. Whilst evidence from small to moderately sized investigations into the effects of the GH/IGF axis on muscle properties and physical performance in humans has been mixed [11], [12], it has been hypothesised that polymorphisms in genes in the GH/IGF axis may contribute to measures of muscle functioning and physical capability, phenotypes that have been shown to be partly heritable [22], [23].

In genome-wide association studies (GWAS), single-nucleotide polymorphisms (SNPs) around *IGF1* have been associated with height [24]–[26] and the C allele of rs35767 upstream of the gene has been associated with increased fasting insulin and insulin resistance risk [27] as well as lower levels of IGF-I [28]–[30]. There is some evidence to suggest that SNPs in *IGF2* are associated with longevity [31], measures of adult body size [32]–[35], grip strength [36], arm and leg strength [37], and post-exercise muscle damage [38], and in a GWAS the A allele of rs7127900 near the gene has been associated with increased risk of prostate cancer [39]. The A allele of functional SNP rs2854744 in the promoter region of *IGFBP3* has been associated with higher levels of IGFBP-3 [18], [40] and may be associated with reduced breast and prostate cancer risk [18]. Increased type 2 diabetes risk has been associated with the C allele of rs2943641 near *IRS1* in GWAS [41]. Studies of the functional SNP rs2665802 (in *GH1*) [42] have found associations between its minor allele and lower colorectal cancer risk [43] and mortality risk in females [44]. The exon-3 deletion of *GHR* (d3GHR) has been associated with response to GH administration in short children [45] and is perfectly tagged by several SNPs in Europeans [46], among which rs6873545 has a suggested association with lung cancer [47]. We therefore examined the associations between these polymorphisms and anthropometric and physical capability traits in 13,364 men and women aged between 52 and 90 years as part of the HALCyon (Healthy Ageing across the Life Course; www.halcyon.ac.uk) programme, in what we believe to be the largest investigation into polymorphisms in the GH/IGF axis and physical capability.

## Methods

### Ethics Statement

Written informed consent was obtained from all participants. Ethical approval for each study was obtained from the relevant ethics committees.

### Study Populations

The Medical Research Council National Survey of Health and Development (NSHD) comprises participants sampled from all births in a week in March 1946 and followed up since. In 1999, at age 53 years, men and women were visited by a research nurse and consent for DNA extraction was given by approximately 2900 members of the cohort. Details of the data collected and the several phases of the study are available on the cohort's website (www.nshd.mrc.ac.uk) and elsewhere [48].

The English Longitudinal Study of Ageing (ELSA) comprises men and women aged 50 years and over who originally participated in the Health Survey for England in 1998, 1999 or 2001. Fieldwork began in 2002–03 (Phase I) with two-yearly follow-ups and in 2004–05 (Phase II) blood samples were provided by 6231 participants. Details of the cohort have been published [49].

The Hertfordshire Cohort Study (HCS) consists of 2997 participants born 1931–39 and registered with a General Practitioner in East, North or West Hertfordshire who attended a clinic in 1994–2004 (Phase I). A second assessment took place in 2004–05 for participants in East Hertfordshire (Phase II). Further details of study design, data collected and summaries of participant characteristics have been published [50] and are available on its website (www.mrc.soton.ac.uk/herts).

The Hertfordshire Ageing Study (HAS) comprises men and women traced in 1994–95, the first follow-up (Phase I), of singleton births from 1920–30 in North Hertfordshire. A total of 717 participants attended a clinic during which DNA was extracted. A second follow-up took place in 2003–05 (Phase II). Details of the recruitment, data collected and summaries of participant characteristics have been described previously [51].

The Boyd Orr cohort is a historical cohort study based on children surveyed in 1937–39 in English and Scottish districts. Participants were followed-up for vital status via the NHS Medical Information Research Service (MIRS) since 1948, with questionnaire administration to survivors in 1997–98 (Phase II) and a research clinic visit in 2002–03 (Phase III), during which DNA was extracted from 728 adults. Details of the study design and the data collected have been described on its website (www.epi.bris.ac.uk/boydorr) and elsewhere [52].

The Caerphilly Prospective Study (CaPS) recruited 2512 men aged between 45 and 59 years in 1979–83 from the town of Caerphilly, South Wales, and its surrounding villages. Blood samples were collected at baseline and at each of the four follow-ups (Phase II: 1984–88, Phase III: 1989–93, Phase IV: 1993–97 and Phase V: 2002–04.) Further details are available on the cohort's website (www.epi.bris.ac.uk/caerphilly/caerphillyprospectivestudy.htm).

The Lothian Birth Cohort 1921 Study (LBC1921) participants were all born in 1921 and completed an IQ assessment at age 11. In 1999–2001 (Phase I) 550 79 year olds, living in and around Edinburgh, attended a clinic. Details of the recruitment into the study are available on its website (www.lothianbirthcohort.ed.ac.uk) and have been published previously [53].

### Genotyping and Quality Control

Genotyping for SNPs rs35767 (−C1245T, *IGF1*), rs7127900 (*IGF2*), rs2854744 (A-202C, *IGFBP3*), rs2943641 (*IRS1*) and rs2665802 (T1663A/T1169A, *GH1*) for all cohorts, except LBC1921, were carried out by KBioscience (www.kbioscience.co.uk). Genotype information for rs7127900 (*IGF2*) and rs2943641 (*IRS1*) in LBC1921 came from a genome-wide scan performed on the Illumina Human610-Quadv1 Chip (www.illumina.com) [54]. The d3GHR (exon-3 deletion of *GHR*) polymorphism resulted from recombination between two near identical retroelements and can therefore be genotyped using a “pseudo-SNP” assay for a single nucleotide difference between the two alleles [55]. Genotyping for the d3GHR pseudo-SNP was performed by KBioscience. Data quality was reviewed by assessing clustering quality (using KBioscience software SNPviewer on their data), call rates and deviation from Hardy-Weinberg equilibrium (HWE). Where deviation from HWE was detected for a study based on the hypothesis test, no exclusions were made due to the high quality of the studies in this investigation and the consistent genotyping methods used for the majority of the polymorphisms across the studies [56].

### Phenotypes

#### Anthropometry

Measurements were conducted either at clinics, during a clinical interview in the home, or from self-report. Body mass index (BMI kg/m^2^) was calculated as weight divided by height squared. Waist-hip ratio (WHR) was defined as waist circumference (cm) divided by hip circumference (cm) and was measured in NSHD, ELSA, HCS, HAS, Boyd Orr and CaPS.

#### Physical Capability and Activity

Grip strength was measured in NSHD, ELSA, HCS, HAS and LBC1921 using electronic or hydraulic dynamometers, with the best measure used in the analysis where more than one trial was conducted. Standing balance tests were conducted in the studies, with participants' eyes open: flamingo [57], (stopped at 30 s) in NSHD, HCS, HAS, Boyd Orr and CaPS, and side-by-side, semi-tandem and full tandem [58] in ELSA. Poor standing balance was defined for this analysis as the inability to complete 5 s. The timed get up and go test [59] was carried out in HCS, HAS, Boyd Orr and CaPS and required participants to get up from a chair, walk 3 m, turn, walk back, turn and sit down. Timed walks over 2.44 m (8 feet) and 6 m were carried out in ELSA and LBC1921 respectively. Speeds were calculated for timed walks and get up and go, with the fastest speeds used in the analysis where more than one trial was conducted. Timed chair rises [60] involved asking participants to rise from a chair and sit back down 5 times in ELSA, HCS and HAS, and 10 times in NSHD; the reciprocal of time taken in seconds ×100 [61] was used in the analysis. Levels of physical activity were derived from self reported levels using questionnaires in NSHD, ELSA, Boyd Orr, CaPS and LBC1921. Individuals were categorised as ‘physically active’ in this analysis if they engaged, at least once a month, in at least moderate sport or activities in NSHD, Boyd Orr, CaPS and LBC1921 or vigorous sport or activities in ELSA.

### Statistical Methods

Where information on ancestry was collected, non-European participants were excluded from the analyses in order to avoid confounding from population stratification [62]. Within studies, linear and logistic regression analyses were conducted on the continuous and dichotomous traits within the cohorts respectively, adjusting for sex in all studies except CaPS, and age in all studies except NSHD and LBC1921. Analyses of physical capability measures were repeated additionally adjusting for height and weight. Due to the low frequency of individuals homozygous for the T allele of rs35767 (n = 296, 2.3%), T allele of rs7127900 (n = 455, 3.5%) and the exon-3 deletion of *GHR* (n = 853, 6.5%), dominant models were used for these polymorphisms in order to avoid the presentation of tables containing cells with very low frequencies in particular cohorts. Additive models were used for rs2854744, rs2943641 and rs2665802 with genotypes coded as 0, 1 and 2 for the number of minor alleles. Likelihood ratio tests were used to compare the fit of the additive models compared with the full genotype model. For continuous traits, the normality of the standardised residuals was inspected with distributional diagnostic plots. Cook's distances [63] were plotted against fitted values, using a cut-off of four divided by sample size, to identify influential outliers in the continuous phenotypes. For the harmonisation of continuous traits that were used to obtain pooled estimates of the genotypic effects, z-score units were calculated in each study by subtracting the study mean and dividing by its standard deviation. The overall mean for z-scores is 0 and standard deviation 1. Beta coefficients calculated on z-score units can be reverted to the original scale by multiplying by an appropriate standard deviation. Two-step [64] meta-analyses using a random-effects model were performed to obtain pooled genotypic effects. The I^2^ measure was used to quantify heterogeneity [65]. Additionally, within-study analyses of the physical capability traits were stratified by physical activity, an indicator shown to modify genotypic effects on anthropometric measures [66] and the effects of rs2665802 on colorectal cancer risk [67]. Finally, the calculation of z-scores, for the continuous traits, and the main analyses were repeated in males and females separately. Quanto [68] was used for power calculations. Reporting of the analyses met the appropriate items of recommended checklists [69], [70]. A two-tailed significance level of p<0.05 was used as evidence of statistical significance. Statistical analysis was performed in Stata 11.1 (StataCorp LP).

## Results

### Cohort Summaries and Genotyping Quality

Relevant genotypic and phenotypic data were available for a total of 13,364 adults aged between 52 and 90 years old (Table 1). The call rates were high, exceeding 95% across the studies for all polymorphisms. The HWE condition was met for rs35767, rs7127900, rs2854744, rs2943641 and rs2665802 in all studies (p-values>0.09) except for rs7127900 in HAS (p-value = 0.001) and rs2665802 in LBC1921 (p-value = 0.02). Whilst the HWE condition was met for d3GHR in ELSA, HAS, Boyd Orr and CaPS (p-values>0.09) it was not met in two of the larger studies, NSHD (p-value = 0.02) and HCS (p-value = 0.04), and was borderline in LBC1921 (p-value = 0.06), with the number of d3/d3 individuals under-represented.

**Table 1:** Summary of Sex, Age and Minor Allele Frequencies by Cohort.

Cohort								
Variable	NSHD	ELSA	HCS	HAS	Boyd Orr	CaPS	LBC1921	Total
Number of participants	2618	5515	2902	551	397	867	514	13364
Male, %	50	46	53	59	45	100	41	52
Age* in years, median (range)	53	65 (52–90+)	66 (59–73)	67 (63–73)	70 (64–82)	73 (65–83)	79 (77–80)	65 (52–90+)
Minor Allele Frequencies								
rs35767 (*IGF1*)	0.16	0.15	0.15	0.16	0.16	0.16	0.14	0.15
rs7127900 (*IGF2*)	0.18	0.18	0.20	0.21	0.19	0.17	0.19	0.19
rs2854744 (*IGFBP3*)	0.47	0.45	0.46	0.45	0.42	0.46	0.42	0.45
rs2943641 (*IRS1*)	0.36	0.35	0.35	0.36	0.38	0.35	0.35	0.35
rs2665802 (*GH1*)	0.41	0.40	0.41	0.41	0.39	0.40	0.41	0.41
d3GHR	0.26	0.27	0.27	0.24	0.27	0.28	0.26	0.27

Numbers of participants represent those with available data for at least one anthropometric or physical capability phenotype and at least one genotype.

*Age at phase from which the majority of variables are taken, i.e. Boyd Orr: III; CaPS: V; ELSA: II; HAS: I; HCS: I; LBC1921: I; NSHD: 1999 Collection. CaPS: Caerphilly Prospective Study; ELSA: English Longitudinal Study of Ageing; HAS: Hertfordshire Ageing Study; HCS: Hertfordshire Cohort Study; NSHD: National Survey of Health and Development.

### Associations between Genotypes and Phenotypes

In ELSA, the study with the widest age range, genotype frequencies in those under 70 years were compared with those over 70 years; the T allele of rs2943641 (*IRS1*) was more common among those over 70 (chi-squared p-value = 0.0059). Associations with age group were not observed for the other polymorphisms (p-values>0.1; data not shown).

**Figure 1:**
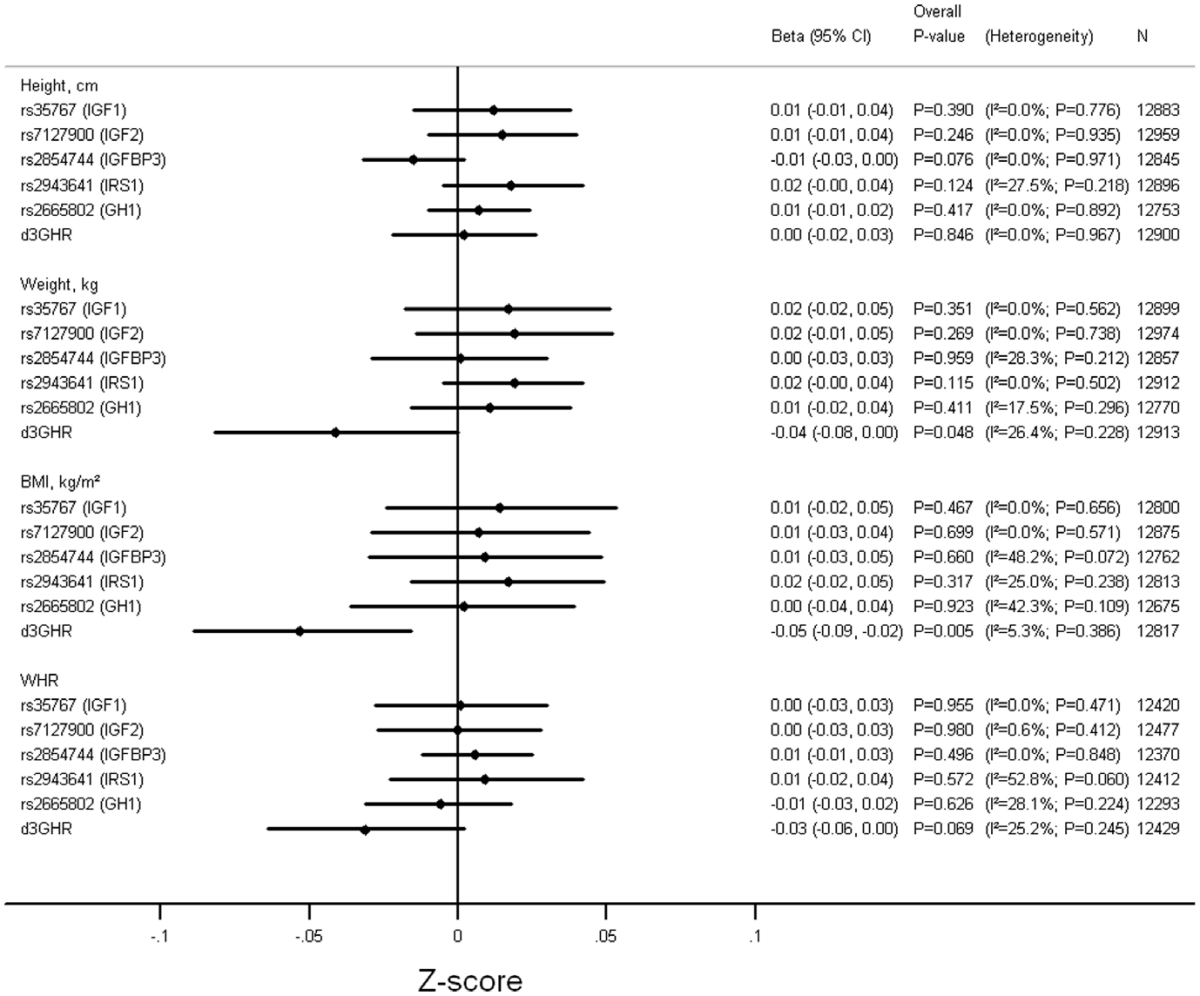
Pooled Results of Associations between Genotypes and Measures of Anthropometry. Adjusted for age and sex. Models used: rs35767 (*IGF1*)- (C/T+T/T) vs. C/C; rs7127900 (*IGF2*)- (C/T+T/T) vs. C/C; rs2854744 (*IGFBP3*- per minor (A) allele; rs2943641 (*IRS1*)- per minor (T) allele; rs2665802 (*GH1*)- per minor (T) allele; d3GHR- (fl/d3+d3/d3) vs. fl/fl. fl: full length; d3: exon-3 deletion.

Figure 1 and Tables S1, S2, S3, S4, S5, S6 show the associations between the polymorphisms and height, weight, BMI and WHR adjusting for age and sex. From the pooled analyses there was evidence for associations between d3GHR and weight (p-value = 0.048) and BMI (p-value = 0.005), with individuals with at least one exon-3 deletion allele having lower weight and BMI compared with individuals retaining both copies of the exon (Figure S1 and S2). There was also a trend among individuals carrying the exon-3 deletion to have a lower WHR (p-value = 0.07) and for rs2854744-A (*IGFBP3*) to be associated with shorter height (p-value = 0.08). There was no evidence for other associations between the polymorphisms and anthropometric measures (p-values>0.1).

**Figure 2:**
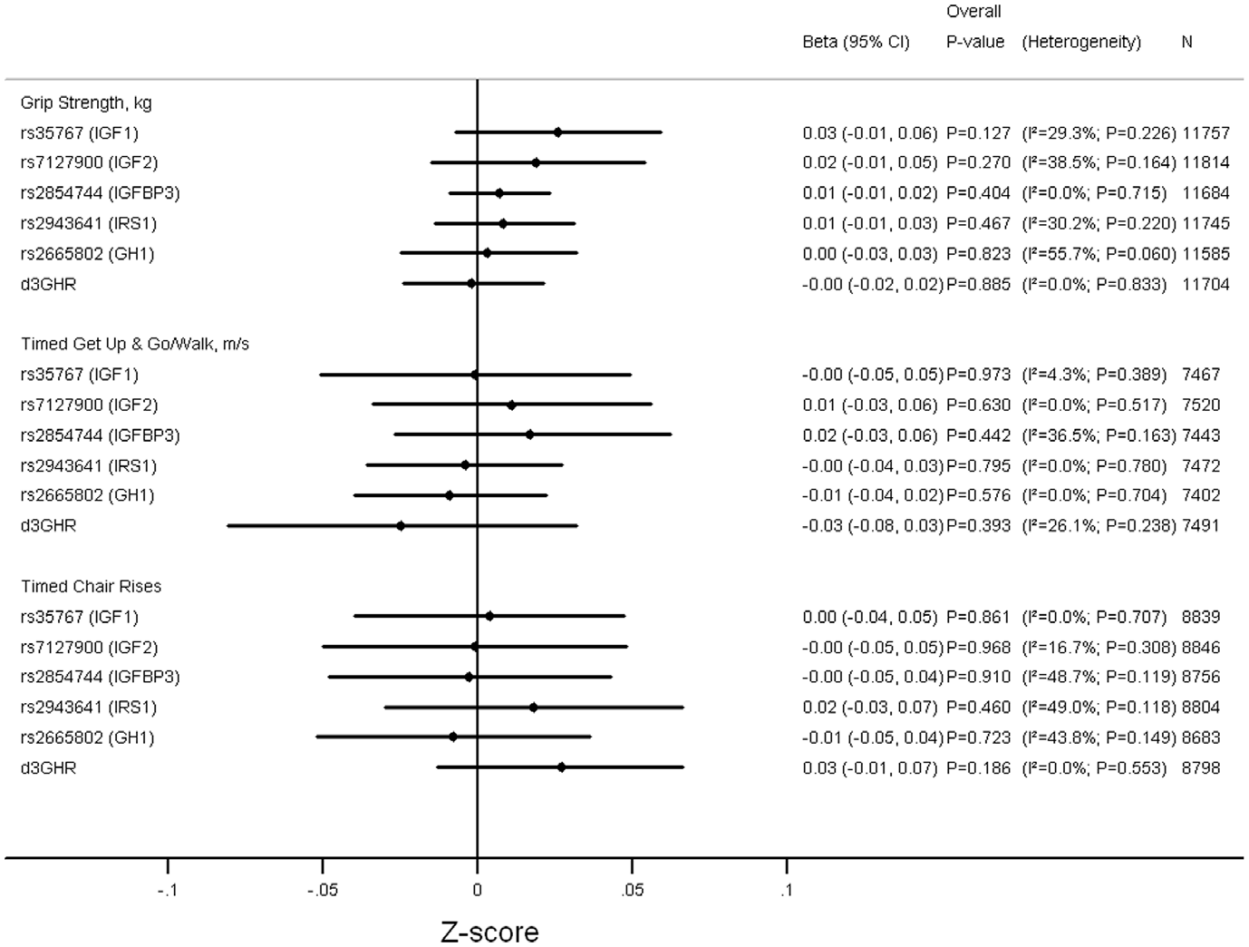
Pooled Results of Associations between Genotypes and Grip Strength, TGUG and Chair Rises. Adjusted for age and sex. Timed chair rises on reciprocal of time taken in sec ×100. Models used: rs35767 (*IGF1*)- (C/T+T/T) vs. C/C; rs7127900 (*IGF2*)- (C/T+T/T) vs. C/C; rs2854744 (*IGFBP3*- per minor (A) allele; rs2943641 (*IRS1*)- per minor (T) allele; rs2665802 (*GH1*)- per minor (T) allele; d3GHR- (fl/d3+d3/d3) vs. fl/fl. fl: full length; d3: exon-3 deletion.

Figure 2 and Tables S1, S2, S3, S4, S5, S6 show that there was no evidence for associations between any of the polymorphisms and grip strength, timed get up and go/walks and timed chair rises after adjusting for age and sex (p-values>0.1). Similarly, there were no associations after additionally adjusting for height and weight (p-values>0.1; data not shown).

**Figure 3:**
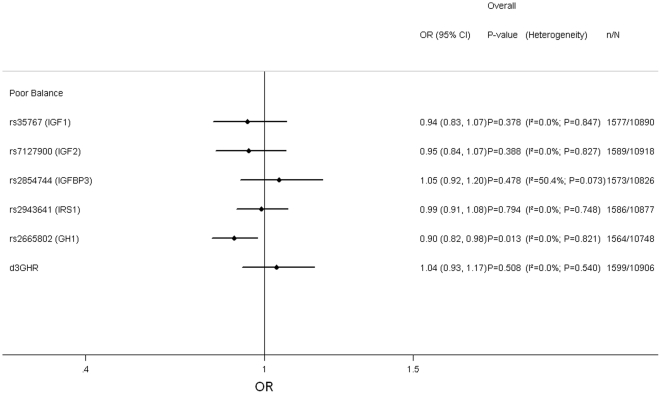
Pooled Results of Associations between Genotypes and Poor Balance. Adjusted for age and sex. Poor balance defined as inability to complete the Flamingo test for 5 s in Boyd Orr, HAS, HCS, NSHD and CaPS, and 5 s of the tandem test in ELSA. Models used: rs35767 (*IGF1*)- (C/T+T/T) vs. C/C; rs7127900 (*IGF2*)- (C/T+T/T) vs. C/C; rs2854744 (*IGFBP3*- per minor (A) allele; rs2943641 (*IRS1*)- per minor (T) allele; rs2665802 (*GH1*)- per minor (T) allele; d3GHR- (fl/d3+d3/d3) vs. fl/fl. fl: full length; d3: exon-3 deletion.

The associations between the polymorphisms and poor balance, adjusting for age and sex, are presented in Figure 3 and Tables S1, S2, S3, S4, S5, S6. There was evidence for an association with SNP rs2665802 (*GH1*) (p-value = 0.01), with the minor allele associated with increased ability to balance (Figure S3). The association remained after additional adjustment for height and weight (p-value = 0.02; data not shown). There was no evidence for associations with the other polymorphisms after adjusting for age, sex (p-values>0.5), height and weight (p-values>0.4; data not shown).

In only a relatively small number of tests did the full genotype model represent a significantly better fit than the per allele model (indicated in Tables S3, S4, S5).

Within NSHD, ELSA, Boyd Orr, CaPS and LBC1921, there was little evidence that the genotypic effects for the physical capability traits differed between the physically active and inactive. Interactions were only observed in Boyd Orr for rs2854744 with balance (p-value = 0.04) and in ELSA for rs2943641 with grip strength (p-value = 0.03); however, the genotypic effects were not significant in either group in ELSA, whilst an association was observed with balance in the physically inactive group in Boyd Orr.

### Investigations by Sex

Figures S4 and S5 present the pooled associations between the polymorphisms and the anthropometric traits adjusting for age in males and females, respectively. There was evidence for a sex difference between rs2943641 (*IRS1*) and height (p-value = 0.03 for heterogeneity between males and females) with the T allele being associated with greater height in females but not males (Figure S6). There was no evidence for any other sex differences between the polymorphisms and anthropometric traits (heterogeneity p-values>0.1).

Pooled associations between the polymorphisms and the physical capability traits, adjusting for age, in males and females are presented in Figures S7 to S9. There was no evidence for sex differences (heterogeneity p-values>0.1), except for rs35767 (*IGF1*) and chair rises (heterogeneity p-value = 0.03) where the genotypic effects were in opposite directions, though not achieving statistical significance in either sex (Figure S10).

## Discussion

We examined associations between six common polymorphisms in the GH/IGF axis and measures of anthropometric and physical capability phenotypes in seven UK cohorts comprising a total of 13,364 adults aged between 52 and 90 years. To our knowledge this is the largest investigation into polymorphisms in the axis and physical capability. The polymorphisms chosen are either functional or have been robustly associated with age-related phenotypes in GWAS. Among the 48 genotype association tests conducted in the main analysis there were few important associations observed. We found evidence for associations between d3GHR and anthropometry, with carriers of at least one exon-3 deletion having lower weight, BMI and a trend towards lower WHR. After adjusting for age and sex we found evidence for an association with physical capability for only one of the polymorphisms, with the minor allele of rs2665802 (*GH1*) being associated with increased ability to balance for at least 5 s. On the whole, the associations for physical capability were similar in males and females, between the physically active and inactive and after additionally adjusting for height and weight. These findings suggest that these polymorphisms are not important contributors to physical capability in older adults.

GWAS in Asian populations have found associations between SNPs around *IGF1*, including rs35767, and height [25], [26]. However, to-date, little evidence has emerged from GWAS supporting highly significant associations between common polymorphisms in the six genes that we investigated in the GH/IGF axis and measures of anthropometry in Europeans [71], [72]. Although, it is possible that SNPs in these six genes may significantly modify the effects on lean body mass of SNPs in other genes that are relevant to the GH/IGF axis [73]. We observed no association between rs35767 (*IGF1*) and measures of body size, consistent with other studies on European populations [27], [74]. A previous investigation in elderly men and women found no consistent associations between rs35767 (*IGF1*) and measures of body composition and walk times, grip strength and timed chair stand [75]. We did not observe poorer measures of physical capability among carriers of the risk allele for prostate cancer for rs7127900 near *IGF2* [39]. Other smaller studies had found no association between rs2854744 (*IGFBP3*) and measures of adult body size in women of European ancestry (n = 1702) [76], or muscle phenotypes in response to strength training in older adults (n = 128) [77]. Unlike a previous study (n = 454) [44], we did not observe associations between rs2665802 (*GH1*) and height in either sex, nor were associations reported with height or BMI elsewhere (sample sizes ranged between 293 and 1003) [43], [78], [79]. Our results suggested that the minor allele of rs2665802 (*GH1*) was associated with better ability to balance for at least 5 s. The allele has also been associated with lower colorectal cancer [43] and mortality risk in females [44], though the null findings observed for the other measures of physical capability would suggest that rs2665802 (*GH1*) does not substantially influence measures of physical capability. In addition, a study of 169 athletes and 155 controls suggests that it is not associated with athletic status [80]. Our meta-analysis provided some evidence for lower weight and BMI among carriers of the exon-3 deletion of *GHR*. Although few specific investigations into the d3GHR polymorphism on population-based studies of adults have been conducted, smaller studies have found no associations with measures of body size (sample sizes ranged between 100 and 831) [46], [81], [82]; however, one study observed associations between its haplotypic block and BMI [46]. We therefore contacted the GIANT Consortium [83] to look up associations between tag SNPs of d3GHR and BMI. No associations were observed in per minor allele models for BMI for any of the five available SNPs, for example, for rs4590183 the data suggested a p-value of 0.98 in an analysis of 123,863 participants, suggesting our observed associations for d3GHR with weight and BMI were false positives.

It is an important finding that this investigation does not provide evidence for a substantial role of these common polymorphisms in the GH/IGF axis and measures of physical capability given the statistical power of this multi-cohort study. Sample size calculations for the quantitative traits for 80% power at the 5% significance level estimated that around 7000 individuals would be required to detect a beta coefficient of 0.07 z-score units under a dominant model for a polymorphism with a MAF of 0.19 or for 0.05 z-score units under an additive model using a MAF of 0.35. For example, there was sufficient power to detect a difference in grip strength of around 0.8 kg under a dominant model for rs7127900 (*IGF2*), assuming a standard deviation of 11. This allows the inference that any associations between the polymorphisms and physical capability traits are very small.

Deviation from the HWE condition was detected for d3GHR in two of the larger studies, NSHD and HCS. However, the only suggested associations for d3GHR were with weight and BMI, which were found to be in opposite directions for these studies (Table S6, Figure S1 and S2), indicating that, although exclusion is not recommended [56], excluding these two studies would not substantially affect the overall pooled results. Furthermore, whilst we observed four deviations from HWE altogether, around two statistically significant tests would be expected given the 42 (6 polymorphisms×7 studies) HWE tests performed.

Whilst the choice of polymorphisms within our candidate genes were primarily based on robust associations observed with age-related traits [18], [27], [39], [41], or functional evidence [42], additional polymorphisms in these genes may be useful for exploring further the influence of common polymorphisms in the GH/IGF axis on measures of physical capability.

### Conclusion

The results of this large, multi-cohort investigation do not support the hypothesis that these common polymorphisms in the GH/IGF axis contribute substantially to objectively measured physical capability in older adults.

## Supporting Information

Figure S1 **Meta-analysis for the Associations between d3GHR Genotype and Weight.** Adjusted for age and sex. Coefficients based on z-scores. fl: full length; d3: exon-3 deletion.
(TIF)

Figure S2 **Meta-analysis for the Associations between d3GHR Genotype and BMI.** Adjusted for age and sex. Coefficients based on z-scores. fl: full length; d3: exon-3 deletion.
(TIF)

Figure S3 **Meta-analysis for the Associations between rs2665802 (** ***GH1*** **) Genotype and Poor Balance.** Adjusted for age, sex. Poor balance defined as inability to complete the Flamingo test for 5 s in Boyd Orr, HAS, HCS, NSHD and CaPS, and 5 s of the tandem test in ELSA.
(TIF)

Figure S4 **Summary of Pooled Results of Associations between Genotypes and Measures of Anthropometry in Males.** Adjusted for age. Models used: rs35767 (*IGF1*)- (C/T+T/T) vs. C/C; rs7127900 (*IGF2*)- (C/T+T/T) vs. C/C; rs2854744 (*IGFBP3*- per minor (A) allele; rs2943641 (*IRS1*)- per minor (T) allele; rs2665802 (*GH1*)- per minor (T) allele; d3GHR- (fl/d3+d3/d3) vs. fl/fl. fl: full length; d3: exon-3 deletion.
(TIF)

Figure S5 **Summary of Pooled Results of Associations between Genotypes and Measures of Anthropometry in Females.** Adjusted for age. Models used: rs35767 (*IGF1*)- (C/T+T/T) vs. C/C; rs7127900 (*IGF2*)- (C/T+T/T) vs. C/C; rs2854744 (*IGFBP3*- per minor (A) allele; rs2943641 (*IRS1*)- per minor (T) allele; rs2665802 (*GH1*)- per minor (T) allele; d3GHR- (fl/d3+d3/d3) vs. fl/fl. fl: full length; d3: exon-3 deletion.
(TIF)

Figure S6 **Meta-analysis for the Associations between rs2943641 (** ***IRS1*** **) Genotype and Height by Sex.** Adjusted for age. Coefficients based on z-scores.
(TIF)

Figure S7 **Summary of Pooled Results of Associations between Genotypes and Grip Strength, Timed Get Up & Go and Chair Rises in Males.** Adjusted for age. Timed chair rises on reciprocal of time taken in sec ×100. Models used: rs35767 (*IGF1*)- (C/T+T/T) vs. C/C; rs7127900 (*IGF2*)- (C/T+T/T) vs. C/C; rs2854744 (*IGFBP3*- per minor (A) allele; rs2943641 (*IRS1*)- per minor (T) allele; rs2665802 (*GH1*)- per minor (T) allele; d3GHR- (fl/d3+d3/d3) vs. fl/fl. fl: full length; d3: exon-3 deletion.
(TIF)

Figure S8 **Summary of Pooled Results of Associations between Genotypes and Grip Strength, Timed Get Up & Go and Chair Rises in Females.** Adjusted for age. Timed chair rises on reciprocal of time taken in sec ×100. Models used: rs35767 (*IGF1*)- (C/T+T/T) vs. C/C; rs7127900 (*IGF2*)- (C/T+T/T) vs. C/C; rs2854744 (*IGFBP3*- per minor (A) allele; rs2943641 (*IRS1*)- per minor (T) allele; rs2665802 (*GH1*)- per minor (T) allele; d3GHR- (fl/d3+d3/d3) vs. fl/fl. fl: full length; d3: exon-3 deletion.
(TIF)

Figure S9 **Summary of Pooled Results of Associations between Genotypes and Poor Balance by Sex.** Adjusted for age. Poor balance defined as inability to complete the Flamingo test for 5 s in Boyd Orr, HAS, HCS, NSHD and CaPS, and 5 s of the tandem test in ELSA. Models used: rs35767 (*IGF1*)- (C/T+T/T) vs. C/C; rs7127900 (*IGF2*)- (C/T+T/T) vs. C/C; rs2854744 (*IGFBP3*- per minor (A) allele; rs2943641 (*IRS1*)- per minor (T) allele; rs2665802 (*GH1*)- per minor (T) allele; d3GHR- (fl/d3+d3/d3) vs. fl/fl. fl: full length; d3: exon-3 deletion.
(TIF)

Figure S10 **Meta-analysis for the Associations between rs35767 (** ***IGF1*** **) Genotype and Timed Chair Rises by Sex.** Adjusted for age. Timed chair rises on reciprocal of time taken in sec ×100. Coefficients based on z-scores.
(TIF)

Table S1 **Anthropometry and Physical Capability by rs35767 (** ***IGF1*** **) Genotype and Cohort.**
(DOCX)

Table S2 **Anthropometry and Physical Capability by rs7127900 (** ***IGF2*** **) Genotype and Cohort.**
(DOCX)

Table S3 **Anthropometry and Physical Capability by rs2854744 (** ***IGFBP3*** **) Genotype and Cohort.**
(DOCX)

Table S4 **Anthropometry and Physical Capability by rs2943641 (** ***IRS1*** **) Genotype and Cohort.**
(DOCX)

Table S5 **Anthropometry and Physical Capability by rs2665802 (** ***GH1*** **) Genotype and Cohort.**
(DOCX)

Table S6 **Anthropometry and Physical Capability by d3GHR Genotype and Cohort.**
(DOCX)
