# Supplementary figures and images for: A Multi-Cohort Study of Polymorphisms in the GH/IGF Axis and Physical Capability: The HALCyon Programme
Source: PLoS One. 2012 Jan 10;7(1):e29883. doi: 10.1371/journal.pone.0029883 (PMC3254646; doi:10.1371/journal.pone.0029883)

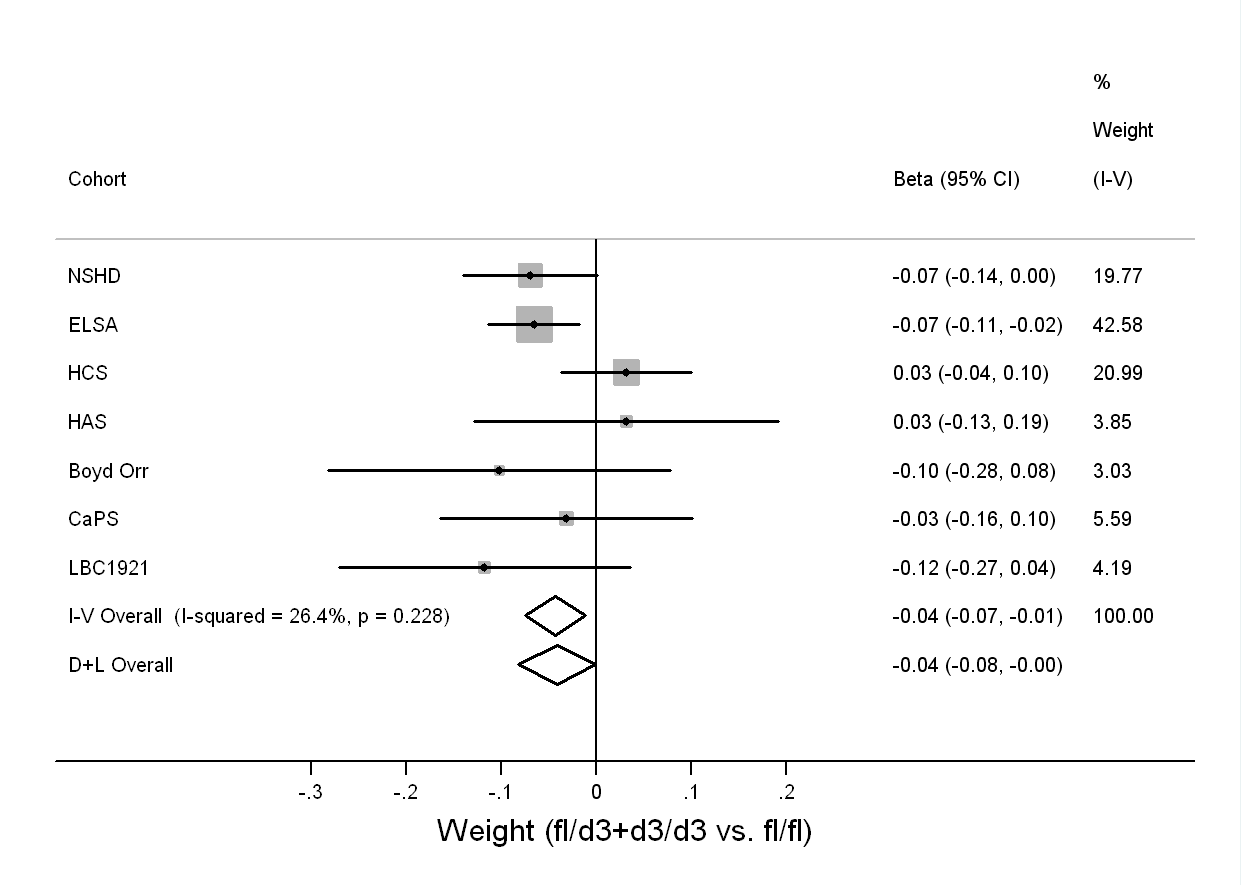

Supplement: Figure S1 — Meta-analysis for the Associations between d3GHR Genotype and Weight. Adjusted for age and sex. Coefficients based on z-scores. fl: full length; d3: exon-3 deletion. (TIF) [file pone.0029883.s001.tif]

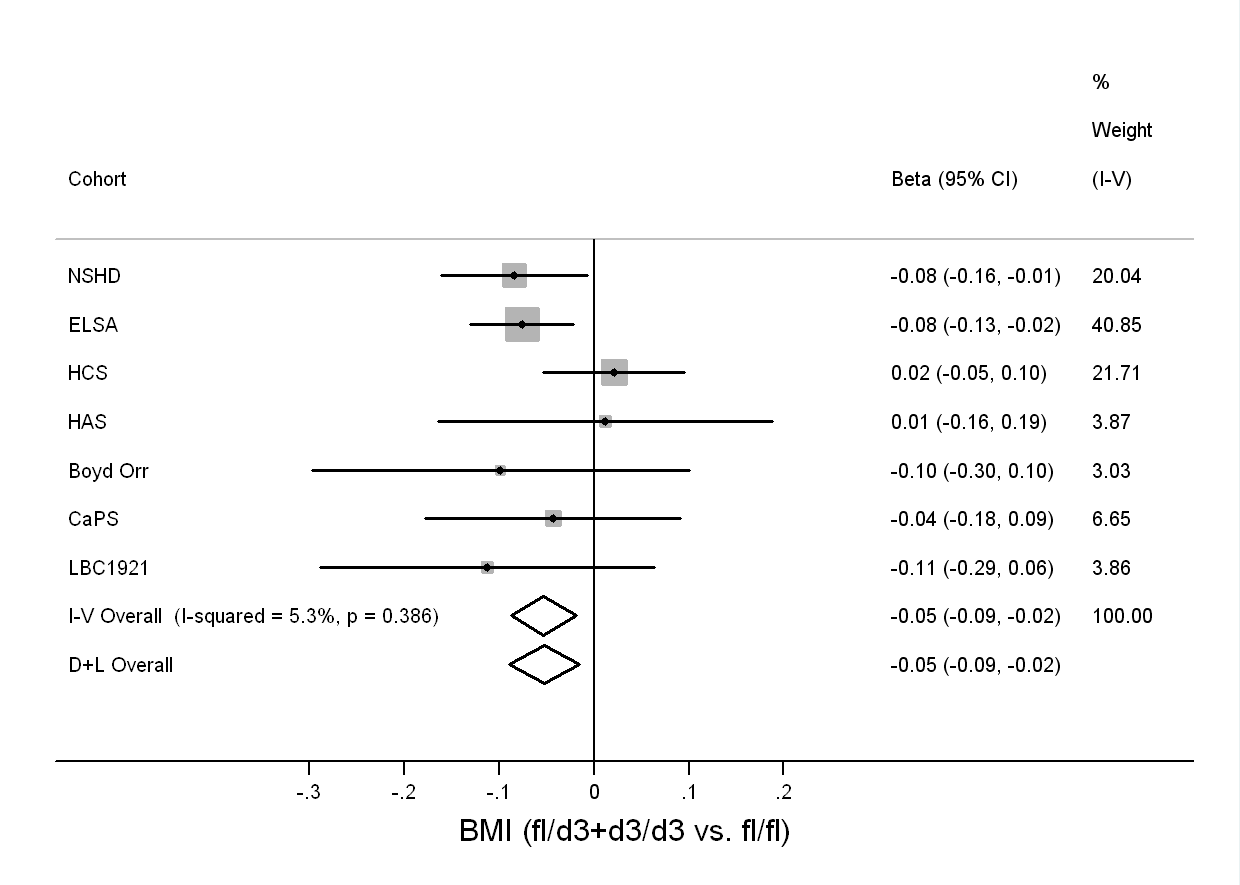

Supplement: Figure S2 — Meta-analysis for the Associations between d3GHR Genotype and BMI. Adjusted for age and sex. Coefficients based on z-scores. fl: full length; d3: exon-3 deletion. (TIF) [file pone.0029883.s002.tif]

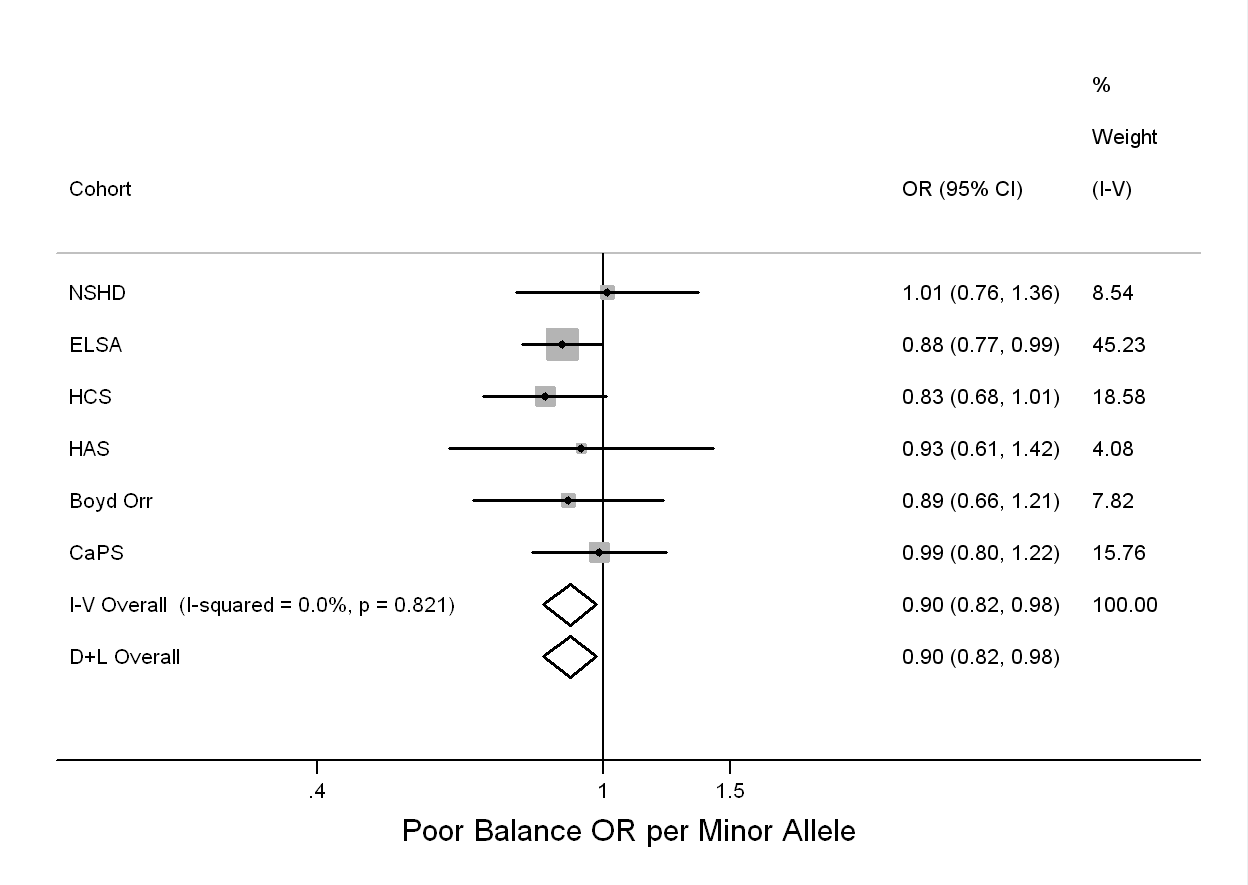

Supplement: Figure S3 — Meta-analysis for the Associations between rs2665802 ( GH1 ) Genotype and Poor Balance. Adjusted for age, sex. Poor balance defined as inability to complete the Flamingo test for 5 s in Boyd Orr, HAS, HCS, NSHD and CaPS, and 5 s of the tandem test in ELSA. (TIF) [file pone.0029883.s003.tif]

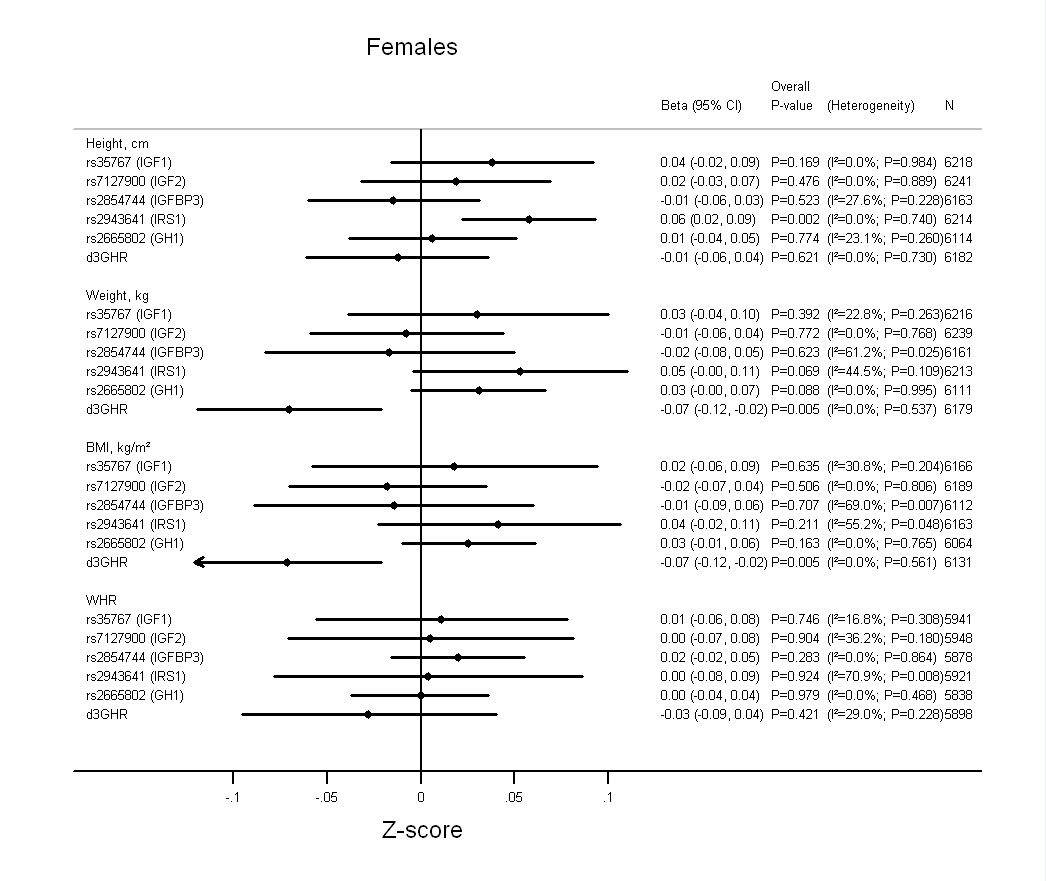

Supplement: Figure S4 — Summary of Pooled Results of Associations between Genotypes and Measures of Anthropometry in Males. Adjusted for age. Models used: rs35767 (IGF1)- (C/T+T/T) vs. C/C; rs7127900 (IGF2)- (C/T+T/T) vs. C/C; rs2854744 (IGFBP3- per minor (A) allele; rs2943641 (IRS1)- per minor (T) allele; rs2665802 (GH1)- per minor (T) allele; d3GHR- (fl/d3+d3/d3) vs. fl/fl. fl: full length; d3: exon-3 deletion. (TIF) [file pone.0029883.s004.tif]

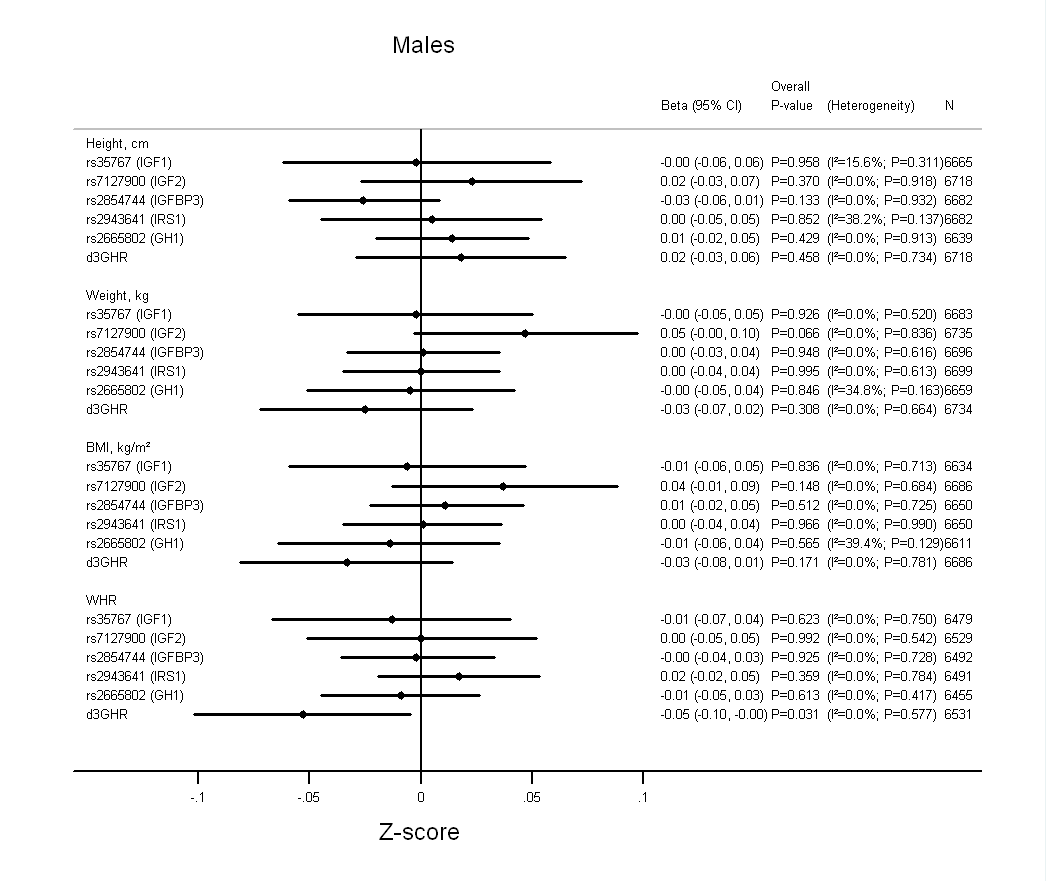

Supplement: Figure S5 — Summary of Pooled Results of Associations between Genotypes and Measures of Anthropometry in Females. Adjusted for age. Models used: rs35767 (IGF1)- (C/T+T/T) vs. C/C; rs7127900 (IGF2)- (C/T+T/T) vs. C/C; rs2854744 (IGFBP3- per minor (A) allele; rs2943641 (IRS1)- per minor (T) allele; rs2665802 (GH1)- per minor (T) allele; d3GHR- (fl/d3+d3/d3) vs. fl/fl. fl: full length; d3: exon-3 deletion. (TIF) [file pone.0029883.s005.tif]

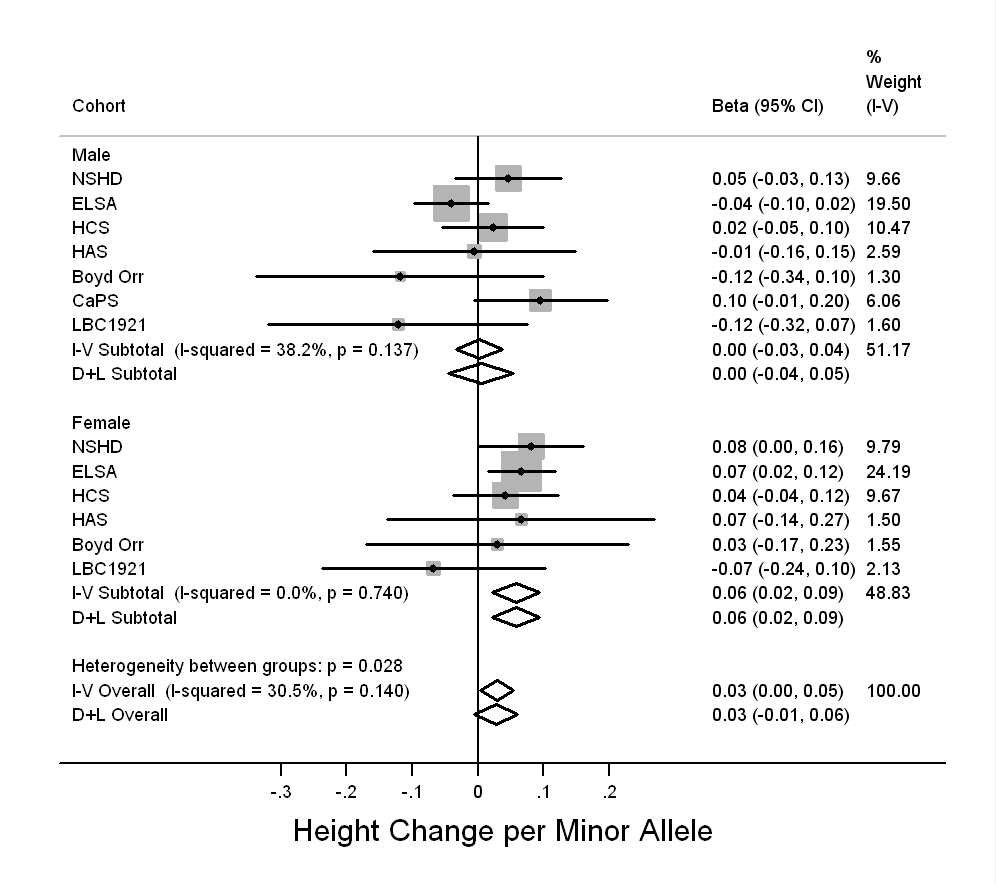

Supplement: Figure S6 — Meta-analysis for the Associations between rs2943641 ( IRS1 ) Genotype and Height by Sex. Adjusted for age. Coefficients based on z-scores. (TIF) [file pone.0029883.s006.tif]

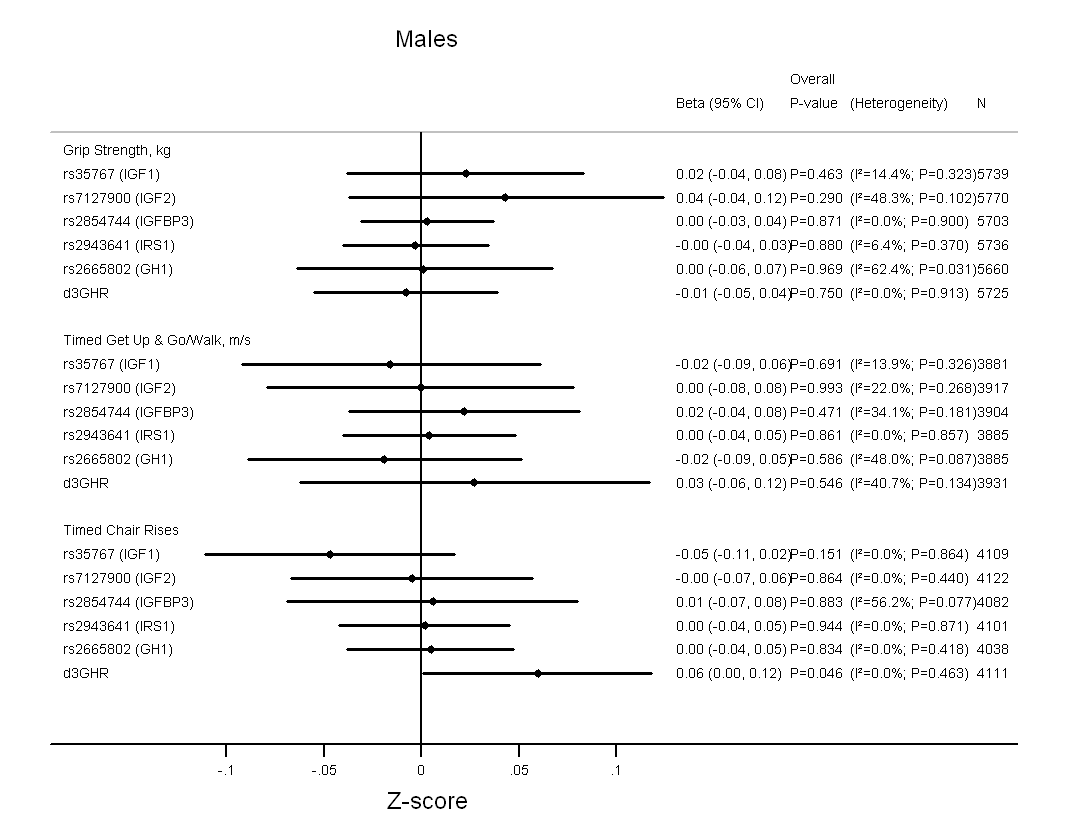

Supplement: Figure S7 — Summary of Pooled Results of Associations between Genotypes and Grip Strength, Timed Get Up & Go and Chair Rises in Males. Adjusted for age. Timed chair rises on reciprocal of time taken in sec ×100. Models used: rs35767 (IGF1)- (C/T+T/T) vs. C/C; rs7127900 (IGF2)- (C/T+T/T) vs. C/C; rs2854744 (IGFBP3- per minor (A) allele; rs2943641 (IRS1)- per minor (T) allele; rs2665802 (GH1)- per minor (T) allele; d3GHR- (fl/d3+d3/d3) vs. fl/fl. fl: full length; d3: exon-3 deletion. (TIF) [file pone.0029883.s007.tif]

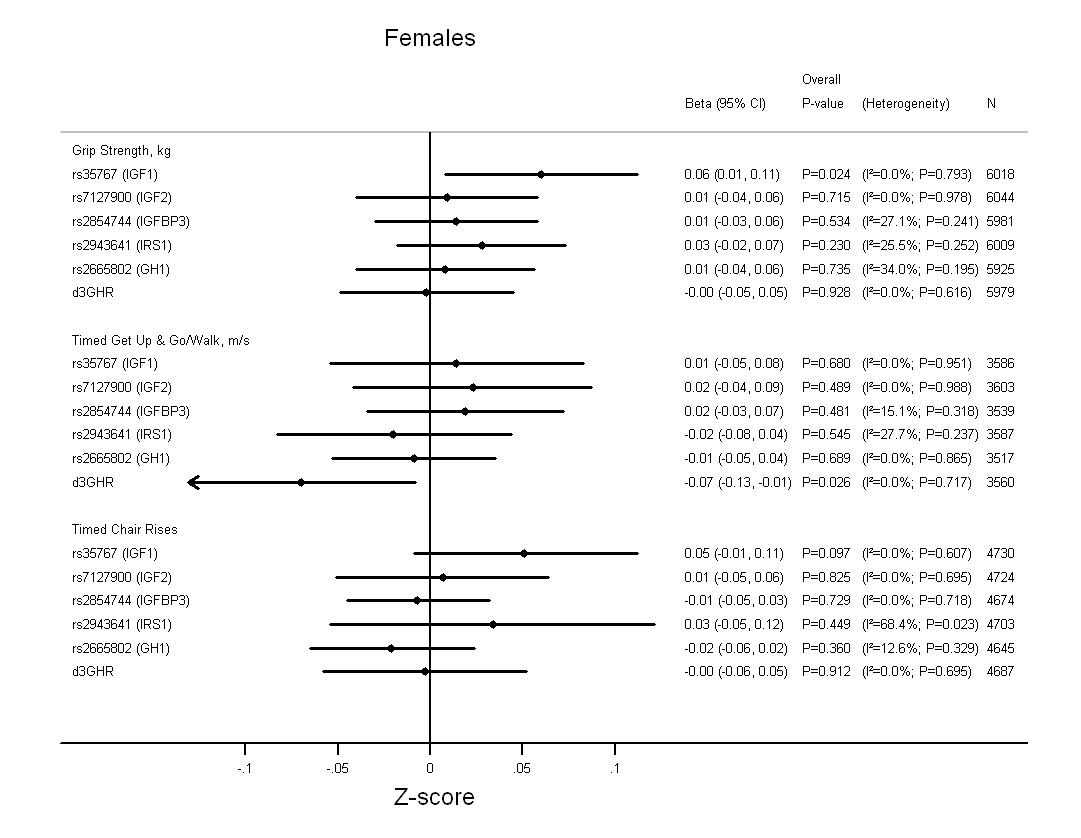

Supplement: Figure S8 — Summary of Pooled Results of Associations between Genotypes and Grip Strength, Timed Get Up & Go and Chair Rises in Females. Adjusted for age. Timed chair rises on reciprocal of time taken in sec ×100. Models used: rs35767 (IGF1)- (C/T+T/T) vs. C/C; rs7127900 (IGF2)- (C/T+T/T) vs. C/C; rs2854744 (IGFBP3- per minor (A) allele; rs2943641 (IRS1)- per minor (T) allele; rs2665802 (GH1)- per minor (T) allele; d3GHR- (fl/d3+d3/d3) vs. fl/fl. fl: full length; d3: exon-3 deletion. (TIF) [file pone.0029883.s008.tif]

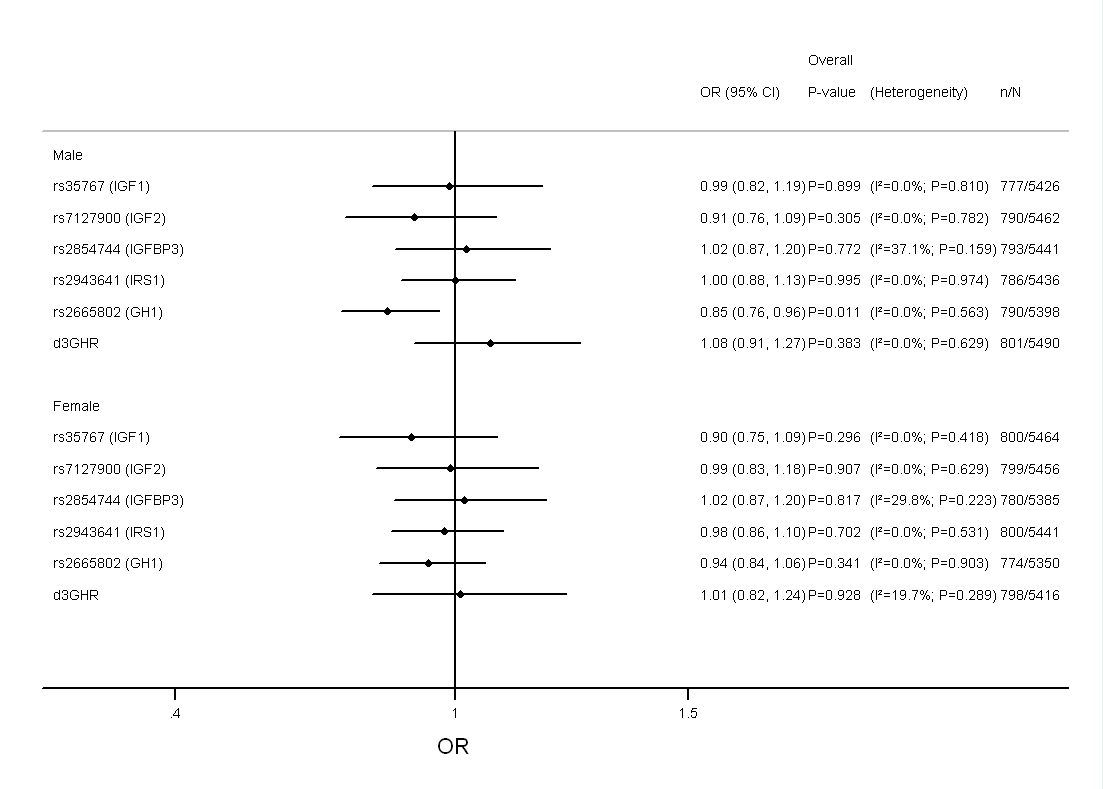

Supplement: Figure S9 — Summary of Pooled Results of Associations between Genotypes and Poor Balance by Sex. Adjusted for age. Poor balance defined as inability to complete the Flamingo test for 5 s in Boyd Orr, HAS, HCS, NSHD and CaPS, and 5 s of the tandem test in ELSA. Models used: rs35767 (IGF1)- (C/T+T/T) vs. C/C; rs7127900 (IGF2)- (C/T+T/T) vs. C/C; rs2854744 (IGFBP3- per minor (A) allele; rs2943641 (IRS1)- per minor (T) allele; rs2665802 (GH1)- per minor (T) allele; d3GHR- (fl/d3+d3/d3) vs. fl/fl. fl: full length; d3: exon-3 deletion. (TIF) [file pone.0029883.s009.tif]

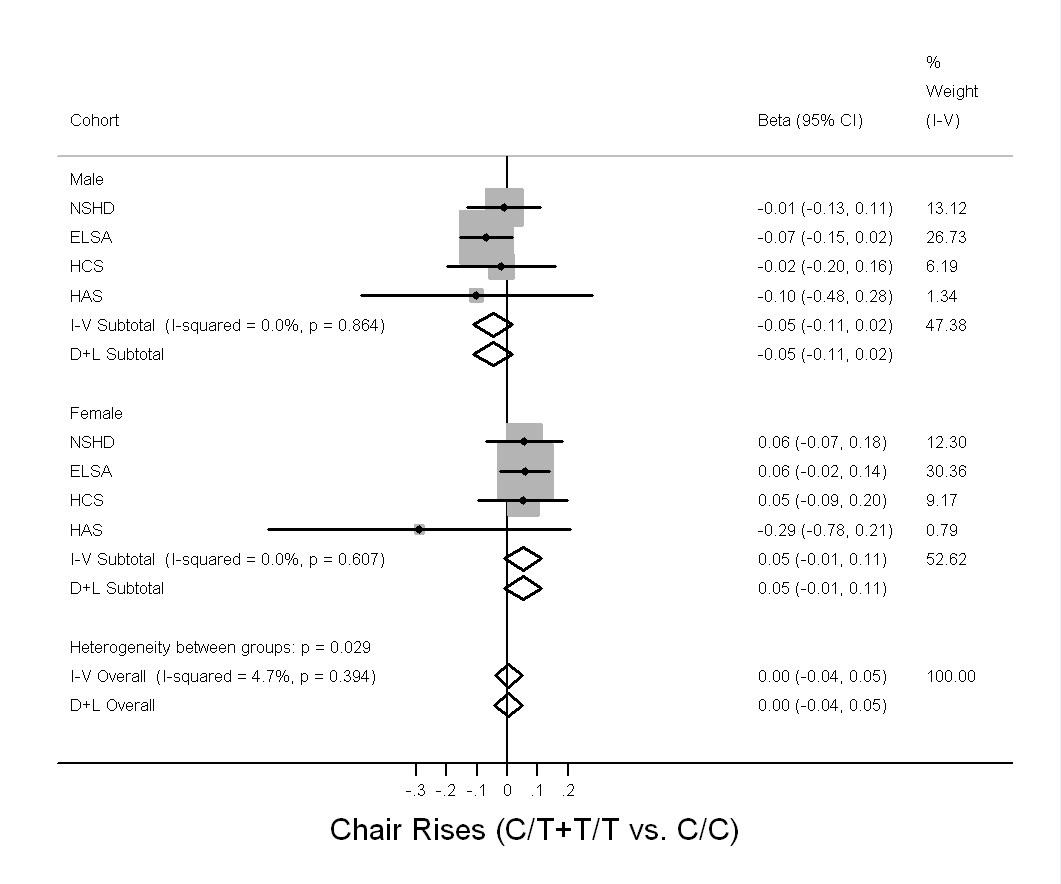

Supplement: Figure S10 — Meta-analysis for the Associations between rs35767 ( IGF1 ) Genotype and Timed Chair Rises by Sex. Adjusted for age. Timed chair rises on reciprocal of time taken in sec ×100. Coefficients based on z-scores. (TIF) [file pone.0029883.s010.tif]
