# Supplementary material for: A Multi-Cohort Study of Polymorphisms in the GH/IGF Axis and Physical Capability: The HALCyon Programme
Source: PLoS One. 2012 Jan 10;7(1):e29883. doi: 10.1371/journal.pone.0029883 (PMC3254646; doi:10.1371/journal.pone.0029883)
Supplement: Table S2 — Anthropometry and Physical Capability by rs7127900 ( IGF2 ) Genotype and Cohort. (DOCX) [file pone.0029883.s012.docx]

**Table S2 Anthropometry and Physical Capability by rs7127900 (*IGF2*) Genotype and Cohort**

| Variable | Cohort | C/C | C/T+T/T | Total | b (95% CI) ^a^ | p-value | Heterogeneity |
| --- | --- | --- | --- | --- | --- | --- | --- |
|  |  | mean (sd) [n] | mean (sd) [n] | mean (sd) [n] |  |  | I^2^%; p-value |
|  | NSHD | 168.3 (8.9) [1725] | 167.9 (9.0) [868] | 168.2 (8.9) [2593] | -0.01 (-0.06- 0.05) | 0.81 |  |
|  | ELSA | 165.7 (9.5) [3553] | 165.7 (9.5) [1753] | 165.7 (9.5) [5306] | 0.02 (-0.02- 0.06) | 0.25 |  |
|  | HCS | 167.8 (9.1) [1829] | 168.1 (9.1) [1007] | 167.9 (9.1) [2836] | 0.02 (-0.03- 0.07) | 0.40 |  |
| Height, cm | HAS | 166.3 (8.9) [345] | 167.5 (8.9) [186] | 166.8 (8.9) [531] | 0.05 (-0.08- 0.17) | 0.45 |  |
|  | Boyd Orr | 164.4 (9.4) [248] | 164.1 (8.9) [135] | 164.3 (9.2) [383] | -0.04 (-0.18- 0.10) | 0.55 |  |
|  | CaPS | 170.2 (6.3) [539] | 170.1 (5.8) [258] | 170.2 (6.2) [797] | -0.01 (-0.15- 0.14) | 0.92 |  |
|  | LBC1921 | 163.0 (9.4) [339] | 163.6 (9.2) [174] | 163.2 (9.3) [513] | 0.02 (-0.10- 0.15) | 0.70 |  |
|  | **Pooled** | [8578] | [4381] | [12959] | 0.015 (-0.010- 0.040) | 0.25 | 0.0; 0.93 |
|  | NSHD | 77.3 (14.6) [1721] | 77.9 (15.3) [865] | 77.5 (14.8) [2586] | 0.06 (-0.01- 0.14) | 0.11 |  |
|  | ELSA | 76.5 (15.3) [3562] | 76.3 (15.2) [1759] | 76.4 (15.3) [5321] | 0.00 (-0.05- 0.05) | 0.97 |  |
|  | HCS | 77.2 (14.1) [1831] | 77.4 (14.3) [1006] | 77.3 (14.2) [2837] | 0.00 (-0.07- 0.08) | 0.90 |  |
| Weight, kg | HAS | 74.3 (13.6) [345] | 75.0 (12.8) [186] | 74.5 (13.3) [531] | -0.00 (-0.17- 0.16) | 0.96 |  |
|  | Boyd Orr | 74.6 (13.6) [248] | 74.2 (14.9) [135] | 74.5 (14.0) [383] | -0.02 (-0.21- 0.17) | 0.86 |  |
|  | CaPS | 80.8 (13.1) [544] | 80.9 (12.0) [258] | 80.8 (12.8) [802] | 0.02 (-0.13- 0.16) | 0.82 |  |
|  | LBC1921 | 69.2 (12.5) [340] | 71.1 (13.2) [174] | 69.8 (12.8) [514] | 0.12 (-0.04- 0.28) | 0.15 |  |
|  | **Pooled** | [8591] | [4383] | [12974] | 0.019 (-0.014- 0.052) | 0.27 | 0.0; 0.74 |
|  | NSHD | 27.2 (4.6) [1720] | 27.6 (4.8) [863] | 27.4 (4.7) [2583] | 0.07 (-0.01- 0.15) | 0.10 |  |
|  | ELSA | 27.8 (4.8) [3503] | 27.7 (4.6) [1730] | 27.8 (4.7) [5233] | -0.02 (-0.08- 0.04) | 0.49 |  |
|  | HCS | 27.4 (4.4) [1829] | 27.3 (4.3) [1006] | 27.4 (4.4) [2835] | -0.01 (-0.09- 0.07) | 0.76 |  |
|  | HAS | 26.8 (4.1) [345] | 26.6 (3.8) [186] | 26.7 (4.0) [531] | -0.04 (-0.22- 0.14) | 0.66 |  |
| BMI, kg/m^2^ | Boyd Orr | 27.6 (4.4) [248] | 27.5 (4.6) [135] | 27.5 (4.5) [383] | 0.00 (-0.21- 0.21) | >0.99 |  |
|  | CaPS | 27.9 (4.1) [539] | 27.9 (3.9) [258] | 27.9 (4.0) [797] | 0.03 (-0.12- 0.18) | 0.70 |  |
|  | LBC1921 | 26.0 (4.1) [339] | 26.5 (4.2) [174] | 26.2 (4.1) [513] | 0.11 (-0.08- 0.29) | 0.25 |  |
|  | **Pooled** | [8523] | [4352] | [12875] | 0.007 (-0.029- 0.044) | 0.70 | 0.0; 0.57 |
|  | NSHD | 0.87 (0.09) [1723] | 0.87 (0.09) [869] | 0.87 (0.09) [2592] | 0.04 (-0.02- 0.10) | 0.18 |  |
|  | ELSA | 0.89 (0.08) [3569] | 0.89 (0.08) [1775] | 0.89 (0.08) [5344] | -0.01 (-0.06- 0.03) | 0.54 |  |
|  | HCS | 0.91 (0.08) [1821] | 0.91 (0.08) [1007] | 0.91 (0.08) [2828] | 0.01 (-0.04- 0.07) | 0.62 |  |
| Waist-hip ratio | HAS | 0.88 (0.08) [344] | 0.88 (0.08) [186] | 0.88 (0.08) [530] | -0.03 (-0.14- 0.08) | 0.56 |  |
|  | Boyd Orr | 0.91 (0.09) [247] | 0.91 (0.09) [135] | 0.91 (0.09) [382] | -0.04 (-0.21- 0.14) | 0.68 |  |
|  | CaPS | 0.93 (0.06) [545] | 0.93 (0.06) [256] | 0.93 (0.06) [801] | -0.11 (-0.26- 0.04) | 0.15 |  |
|  | **Pooled** | [8249] | [4228] | [12477] | 0.000 (-0.027- 0.028) | 0.98 | 0.6; 0.41 |
|  | NSHD | 38.1 (14.4) [1664] | 37.3 (14.2) [840] | 37.8 (14.3) [2504] | -0.02 (-0.07- 0.04) | 0.61 |  |
|  | ELSA | 32.0 (11.4) [3641] | 32.2 (11.8) [1788] | 32.0 (11.5) [5429] | 0.04 (0.01- 0.07) | 0.0193 |  |
| Grip strength, kg | HCS | 35.6 (10.9) [1831] | 36.2 (11.4) [1007] | 35.8 (11.0) [2838] | 0.04 (-0.01- 0.08) | 0.12 |  |
|  | HAS | 31.2 (9.6) [345] | 32.7 (10.5) [185] | 31.7 (9.9) [530] | 0.05 (-0.06- 0.17) | 0.35 |  |
|  | LBC1921 | 26.6 (9.3) [339] | 26.2 (8.9) [174] | 26.5 (9.1) [513] | -0.09 (-0.21- 0.03) | 0.15 |  |
|  | **Pooled** | [7820] | [3994] | [11814] | 0.019 (-0.015- 0.054) | 0.27 | 38.5; 0.16 |
| Timed 2.44m walk, m/s | ELSA | 0.93 (0.30) [2306] | 0.93 (0.30) [1176] | 0.93 (0.30) [3482] | 0.01 (-0.05- 0.07) | 0.78 |  |
| Timed 3m get up and go, m/s | HCS | 0.57 (0.10) [1413] | 0.57 (0.10) [766] | 0.57 (0.10) [2179] | 0.02 (-0.07- 0.10) | 0.73 |  |
| Timed 3m get up and go, m/s | HAS | 0.50 (0.11) [138] | 0.52 (0.13) [72] | 0.51 (0.12) [210] | 0.17 (-0.10- 0.44) | 0.23 |  |
| Timed 3m get up and go, m/s | Boyd Orr | 0.67 (0.15) [248] | 0.67 (0.16) [135] | 0.67 (0.16) [383] | 0.04 (-0.16- 0.24) | 0.68 |  |
| Timed 3m get up and go, m/s | CaPS | 0.60 (0.13) [508] | 0.60 (0.13) [248] | 0.60 (0.13) [756] | 0.05 (-0.10- 0.19) | 0.53 |  |
| Timed 6m walk, m/s | LBC1921 | 1.42 (0.37) [336] | 1.37 (0.37) [174] | 1.40 (0.37) [510] | -0.14 (-0.31- 0.04) | 0.14 |  |
|  | **Pooled** | [4949] | [2571] | [7520] | 0.011 (-0.034- 0.056) | 0.63 | 0.0; 0.52 |
| Timed chair rises^b^-10 rises | NSHD | 5.20 (1.67) [1618] | 5.18 (1.72) [808] | 5.20 (1.68) [2426] | -0.01 (-0.09- 0.08) | 0.84 |  |
| Timed chair rises^b^-5 rises | ELSA | 9.62 (3.15) [3166] | 9.62 (3.18) [1556] | 9.62 (3.16) [4722] | 0.01 (-0.04- 0.07) | 0.60 |  |
| Timed chair rises^b^-5 rises | HCS | 6.26 (1.46) [976] | 6.19 (1.50) [525] | 6.23 (1.48) [1501] | -0.06 (-0.16- 0.04) | 0.26 |  |
| Timed chair rises^b^-5 rises | HAS | 5.27 (1.55) [132] | 5.59 (1.48) [65] | 5.37 (1.53) [197] | 0.21 (-0.08- 0.50) | 0.16 |  |
|  | **Pooled** | [5892] | [2954] | [8846] | -0.001 (-0.050- 0.048) | 0.97 | 16.7; 0.31 |
|  |  |  |  |  |  |  |  |
| Variable | Cohort | C/C | C/T+T/T | Total | OR (95% CI)^a^ | p-value | Heterogeneity |
|  |  | n (%)^c^ | n (%)^c^ | n (%)^c^ |  |  | I^2^%; p-value |
| Balance <5s-One legged | NSHD | 69 (4.1) | 32 (3.8) | 101 (4.0) | 0.91 (0.59- 1.39) | 0.65 |  |
| Balance <5s-Tandem | ELSA | 472 (12.9) | 241 (13.3) | 713 (13.0) | 0.97 (0.80- 1.16) | 0.71 |  |
| Balance <5s-Flamingo | HCS | 185 (18.4) | 91 (16.7) | 276 (17.8) | 0.87 (0.66- 1.15) | 0.33 |  |
| Balance <5s-Flamingo | HAS | 40 (29.0) | 26 (35.6) | 66 (31.3) | 1.35 (0.73- 2.51) | 0.34 |  |
| Balance <5s-Flamingo | Boyd Orr | 94 (38.1) | 55 (40.7) | 149 (39.0) | 1.05 (0.68- 1.63) | 0.83 |  |
| Balance <5s-Flamingo | CaPS | 194 (37.7) | 90 (36.1) | 284 (37.2) | 0.88 (0.63- 1.21) | 0.43 |  |
|  | **Pooled**^d^ | [1054/7257] | [535/3661] | [1589/10918] | 0.95 (0.84- 1.07) | 0.39 | 0.0; 0.83 |

a: Beta coefficients or odds ratios based on z-scores for (C/T+T/T) vs. C/C adjusted for age and sex.

b: Reciprocal of time taken in sec x 100.

c: #participants unable to balance for at least 5s (%).

d: Pooled: [#participants unable to balance for at least 5s /total # participants with relevant data].

WHR in CaPS from Phase III. Balance, timed walk and chair rises in HCS from both phases. Balance, timed get up and go and chair rises in HAS from Phase II.
